# *In Vitro *assessment of the nutritive value of expanded soybean meal for dairy cattle

**DOI:** 10.1186/2049-1891-3-10

**Published:** 2012-03-20

**Authors:** Eman A Elwakeel, Evan C Titgemeyer, Zongjia J Cheng, Abdelaziz M Nour, Mohamed EA Nasser

**Affiliations:** 1 Department of Animal & Fish Production, Faculty of Agriculture, Alexandria University, Alexandria 21545, Egypt; 2 Department of Animal Sciences and Industry, Kansas State University, Manhattan, KS 66506, USA; 3 American Soybean Association-International Marketing, Beijing, China

**Keywords:** Digestibility, Escape, Lysine, Protein, Rumen

## Abstract

Little information is available about the nutritive value of expanded soybean meal, which is produced by expansion of soybeans prior to solvent extraction of the oil. During processing, expanded soybean meal is subjected to additional heat, which might increase the concentration of ruminally undegraded protein. Processing of soybeans with heat during oil extraction could affect lysine availability by increasing ruminally undegraded protein or by impairing intestinal digestion. Our objective was to compare solvent and expanded soybeans with regard to chemical composition and nutritive value for dairy cattle. Samples of expanded soybean meal (n = 14) and solvent-extracted soybean meal (n = 5) were obtained from People's Republic of China to study effects of the expansion process on nutritive value for dairy cattle. Solvent-extracted soybean meal (n = 2) and mechanically extracted (heated) soybean meal (n = 2) from the United States served as references for comparison. Samples were analyzed for crude fat, long-chain fatty acids, crude protein, amino acids, chemically available lysine, *in situ *ruminal protein degradation, and *in vitro *intestinal digestibility. No differences were found between solvent-extracted soybean meals from China and expanded soybean meals from China for crude fat, crude protein, amino acids, or chemically available lysine. *In situ *disappearance of nitrogen, ruminally undegraded protein content, and *in vitro *intestinal digestion of the ruminally undegraded protein were generally similar between solvent-extracted soybean meals made in China and expanded soybean meals made in China; variation among soybean meals was small. Results indicate that the additional heat from the expansion process was not great enough to affect the nutritive value of soybean meal protein for ruminants. Although expansion may improve the oil extraction process, the impact on the resulting soybean meal is minimal and does not require consideration when formulating ruminant diets.

## Introduction

Soybean meal (SBM), a good source of lysine, is widely used as a protein supplement for ruminants [1]. Due to extensive ruminal degradation of protein in SBM, research has focused on treating SBM to increase the ruminally undegraded protein (RUP) content [2].

During solvent extraction of soybeans, the whole soybean is cracked and heated to approximately 60 to 74°C, then flaked and exposed to hexane solvent to extract the oil [3,4]. After the hexane is volatilized, SBM is heated to destroy anti-trypsin factors. For production of expanded SBM, the soybeans are heated by friction during passage through the expander; steam injection may also be employed. Upon exit through the expander die at temperatures typically near 120°C, the sudden drop in pressure expands the product, which destroys cell structure and improves oil extraction and lecithin recovery while reducing the amount of solvent needed [5,6]. After cooling, the expanded product is extracted with hexane, which is similar to solvent extraction of soybeans [5].

The production of expanded SBM is increasingly popular in People's Republic of China, and this has led to a need for information related to its nutritive value, particularly for dairy cattle that could benefit from products with greater concentrations of RUP. Because expanded SBM is subjected to additional heat, it was expected to have greater RUP than solvent-extracted SBM. Processing of soybeans with heat during oil extraction could increase the availability of lysine by increasing RUP or could decrease lysine availability by impairing intestinal digestion. No research is available on the effect of expanded SBM processing on ruminal protein degradability. Douglas and Parsons [7] evaluated the nutritive value of expanded SBM for poultry by measuring its amino acid (AA) content and the protein solubility and also studied the effect of feeding expanded SBM to broiler chicks. They found that the nutritive value of the expanded SBM was similar to standard solvent-extracted SBM.

Our objective was to compare solvent and expanded SBM with regard to chemical composition, chemical lysine availability, *in situ *ruminal protein degradability, and intestinal protein digestibility of SBM.

## Materials and methods

Procedures for these experiments were approved by the Kansas State University Institutional Animal Care and Use Committee.

### Sources of SBM samples

Twenty-one samples of SBM were obtained from People's Republic of China to study effects of the expansion process on nutritive value for dairy cattle. Among the 21 SBM samples, 14 were expanded SBM (C-eSBM), five were typical solvent-extracted SBM (C-sSBM), and two were expanded full-fat soybeans (C-ff-eSB). The samples were produced at different oil-extraction facilities located throughout China. Specific processing methods were not available for individual samples, and the sources of soybeans were also unknown. Four SBM samples were obtained from the United States to serve as references for methodological validation, and these included two solvent-extracted SBM (US-sSBM) and two mechanically extracted (heated) SBM (US-meSBM).

### Chemical composition of SBM

All SBM samples were analyzed for dry matter (105°C in a forced oven for 24 h), ash (450°C in ash oven for 16 h), crude fat (method number 920.39) [8], crude protein (N × 6.25; Kjeldahl procedure), long-chain fatty acids, and AA. Fatty acids were measured by gas chromatography analysis of fatty acid methyl esters [9] using a capillary column (100 m × 0.25 mm × 0.20 μm film) of Supelco SP-2560 (Sigma-Aldrich, St. Louis, MO) with a helium gas flow rate of 1 mL/min. Amino acids were measured by cation-exchange HPLC with *o*-phthalaldehyde post-column derivatization following hydrolysis in 6 mol/L HCl for 24 h at 105°C.

### Lysine chemical availability

Available lysine was measured as lysine residues that reacted with 1-fluro-2,4-dinitrobenzene (FDNB) as described by Carpenter [10]. In brief, samples were mixed with 8% NaHCO_3 _and reacted with FDNB solution (12 mL FDNB dissolved in 450 mL ethanol) for 2 h at 20°C to yield dinitrophenyl-lysine resides from lysine residues with reactive amino groups. Following evaporation of ethanol, HCl was added to yield a final concentration of 6 mol/L and samples were hydrolyzed 105°C for 16 h. A portion of each filtered sample was extracted 3 times with diethyl ether to remove interfering compounds and brought to volume with 1 mol/L HCl. For blanks, another portion of the filtrate was extracted once with diethyl ether, reacted with methoxycarbonyl chloride to yield an ether-soluble compound from lysine, then acidified with HCl and extracted 3 times with diethyl ether before being brought to volume with water. Absorbance was measured with a plate reader (PowerWave XS; BioTek, Winooski, VT) at 435 nm. Standards were dinitrophenyl-lysine, and correction for incomplete recovery of dinitrophenyl-lysine was as described by Awawdeh et al. [11].

### *In situ *protein degradability of SBM

All SBM samples were ground to pass through a 2-mm screen (Wiley Mill; Arthur H. Thomas Co., Philadelphia, PA), and 2-g samples were weighed into Dacron bags (5 cm × 10 cm; 53 ± 10 μm pore size; Ankom Technology, Macedon, NY) to provide a weight/surface ratio of 20 mg/cm^2^. Bags were heat-sealed and placed in 24 weighted mesh bags (36 cm × 42 cm; 26 Dacron bags/mesh bag), with each mesh bag representing a single fermentation time point (0, 4, 8, 16, 24, or 48 h). Blank Dacron bags were included for each time point. Bags were presoaked in cold tap water for 15 min before placing all of them (except 0 h) in the ventral sacs of the rumen of four ruminally cannulated lactating Holstein cows fed a typical dairy diet (Table 1), then each of the six mesh bags per cow was removed at the determined time point. After they were removed from the rumen, bags were placed immediately in cold tap water, coarsely rinsed for about 15 min, and transferred to the laboratory, where they were washed with a top-loading washing machine (Kenmore Ultra Fabric Care; Sears, Roebuck and Co., Chicago, IL) using cold water for five cycles [12] with 1 min of agitation and 2-min spins [13]. The bags were dried at 55°C for 24 h, then air-equilibrated overnight. The nitrogen (N) content of each bag plus residues was determined using the Kjeldahl method.

**Table 1:** Composition of diet fed to cows used for *in situ *fermentations

Ingredient	% of dry matter
Alfalfa hay	18.70
Corn silage	21.21
Wet corn gluten feed	31.03
Ground corn grain	10.67
Whole cotton seed	4.68
Ground sorghum grain	4.62
Mechanically extracted soybean meal	4.32
Fish meal	0.31
Cane molasses	0.44
Fat (Megalac-R; Church and Dwight, Princeton, NJ)	0.84
Limestone	1.38
Sodium bicarbonate	1.03
Salt	0.14
Magnesium oxide	0.16
Trace mineral premix^1^	0.05
Selenium premix, 600 ppm^2^	0.03
Vitamin premix^3^	0.19
Ethylenediamine dihydriodide^4^	+
Rumensin^5^	0.01
Yeast^6^	0.19

^1 ^Zinpro 4-plex (Zinpro, Eden Prairie, MN). Contained zinc methionine, manganese methionine, copper lysine, and cobalt glucoheptonate. Provided to final diet (mg/kg dry matter): Zn, 14; Mn, 8; Cu, 5; Co, 1

^2 ^Provided 0.19 mg Se/kg diet dry matter as sodium selenite

^3 ^Provided 3,600 IU vitamin A, 1,600 IU vitamin D, and 76 IU vitamin E per kg diet dry matter

^4 ^Provided 0.38 mg ethylenediamine dihydriodide/kg diet dry matter

^5 ^Provided 11 mg monensin/kg diet dry matter (Elanco Animal Health, Greenfield, IN)

^6 ^Culture of *Saccharomyces cerevisiae *(Diamond V XP; Cedar Rapids, IA)

The undegradability of protein *in situ *was modeled for each SBM source for each of the four cows separately using the NLIN procedure of SAS (SAS Inst. Inc., Cary, NC) with a model of: Residual N = C+(B × e^-(Kd × t)^), where C = fraction completely resistant to degradation, B = potentially degradable fraction, Kd = fractional rate of disappearance of B, and t = time. Because analysis of most SBM samples within most of the cows yielded estimates of C = 0, the model was modified to: Residual N = B × e^-(Kd × t)^. The RUP was calculated as: RUP = B × (Kp/(Kp+Kd)), where Kp were assumed passage rates of 0.04, 0.06, or 0.08/h.

### Intestinal digestibility of ruminally undegradable protein (three-step procedure)

Intestinal protein digestibility was determined using the three-step *in vitro*/enzymatic procedure of Calsamiglia and Stern [14]. Dacron bags containing SBM samples were prepared, incubated *in situ *for 16 h, and processed as described above. Each sample was incubated in 20 Dacron bags with 5 bags from each SBM sample being incubated in each of 4 cows. Residuals were removed from the bags by cutting off the top of the bags and removing residues by hand. Residues of SBM were composited by cow (5 bags for each SBM sample) and ground using a mortar and pestle. The N content of the residues was determined using the Kjeldahl method, and these values were used to determine the sample size for measuring intestinal protein digestibility as described by Calsamiglia and Stern [14]. In brief, samples (15 mg N) were incubated in 10 mL of pepsin (P-7012, Sigma, St. Louis, MO, USA) solution (1 g/L adjusted to pH 1.9 with 0.1 mol/L HCl) at 38°C for 1 h. Then, 0.5 mL of 1 mol/L NaOH was added, followed by addition of 13.5 mL of pancreatin (P-7545, Sigma) solution (3 g/L pancreatin and 50 mg/L thymol in KH_2_PO_4 _buffer solution adjusted to pH 7.8) and incubation at 38°C for 24 h. Then, 3 mL of trichloroacetic acid (100% wt/vol) was added to stop the enzymatic reaction and precipitate undigested protein. Following centrifugation at 10,000 × g for 15 min, supernatants were analyzed for N by the Kjeldahl procedure.

### Statistical analyses

The fraction of N remaining at each time point was analyzed statistically as a complete block design using the Mixed procedure of SAS (SAS Inst., Inc., Cary, NC). The model contained the effect of cow (blocking factor), time, SBM classification, and the SBM × time interaction. Observations for B pool size, Kd, RUP, intestinal digestibility, and total N availability were analyzed statistically with the Mixed procedure of SAS for a complete block design, with cow serving as the blocking factor. Chemical compositions were analyzed as for a completely randomized design with SBM classification included in the model. Means were separated using pair-wise *t*-tests when the *F*-test was significant (*P *< 0.05).

## Results

### Chemical composition of SBM

Nutrient compositions of SBM samples obtained from China and the United States are presented in Table 2. Organic matter content was greatest for the C-ff-SB and least for US-sSBM (*P *< 0.05). Crude fat differed (*P *< 0.01) among SBM products; C-ff-SB had the highest value (189 g/kg) followed by US-meSBM (65 g/kg), but no difference was measured between C-sSBM and C-eSBM for their crude fat content or their long-chain fatty acid profile. The C-ff-SB and US-meSBM contained a lower proportion of long-chain fatty acids as C16:0 and a higher proportion as C18:1 compared with other SBM products (*P *< 0.01).

**Table 2:** Composition of soybean meal (SBM) samples from China and the United States

	SBM made in China			United States SBM		
**Item^1^**	**C-sSBM**	**C-eSBM**	**C-ff-eSB**	**US-sSBM**	**US-meSBM**	**SEM^2^**
n	5	14	2	2	2	
Organic matter	930^b^	929^b^	943^a^	921^c^	932^b^	0.60
Crude fat	15^c^	9^c^	189^a^	9^c^	65^b^	2.6
Total fatty acids	16^c^	14^c^	115^a^	14^c^	41^b^	1.1
C16:0, g/kg FA	175^a^	177^a^	121^b^	172^a^	125^b^	3.7
C18:0, g/kg FA	45	45	46	44	45	0.8
C18:1, g/kg FA	118^b^	114^b^	187^a^	107^b^	166^a^	5.0
C18:2, g/kg FA	524	526	523	541	530	2.4
C18:3, g/kg FA	97	94	90	95	101	4.1
CP	482^ab^	490^a^	387^c^	503^a^	450^b^	5.2
Amino acids						
Aspartic acid	53.6^ab^	54.4^a^	44.5^c^	54.3^ab^	49.5^bc^	0.77
Threonine	19.8^ab^	20.2^a^	16.2^c^	20.8^a^	18.7^b^	0.22
Serine	26.1^a^	26.6^a^	21.4^c^	27.3^a^	24.0^b^	0.27
Glutamic acid	86.8^a^	89.1^a^	71.3^b^	91.6^a^	79.2^b^	1.10
Glycine	21.7^ab^	22.1^a^	17.7^c^	22.9^a^	20.2^b^	0.24
Alanine	21.0^ab^	21.5^a^	17.2^c^	21.9^a^	19.8^b^	0.24
Valine	21.3^a^	21.9^a^	17.4^b^	22.1^a^	20.2^a^	0.30
Methionine	3.9^ab^	4.1^a^	3.6^b^	4.0^ab^	3.6^b^	0.06
Isoleucine	23.9^ab^	24.5^a^	19.8^c^	24.9^a^	22.4^b^	0.30
Leucine	36.6^ab^	37.5^a^	30.2^c^	38.9^a^	34.5^b^	0.43
Tyrosine	15.2^a^	15.6^a^	13.3^b^	16.1^a^	14.6^ab^	0.22
Phenylalanine	25.4^ab^	25.9^a^	21.0^c^	26.2^a^	23.4^bc^	0.34
Histidine	11.6^a^	11.9^a^	9.9^b^	11.7^a^	11.2^a^	0.15
Lysine	33.8^ab^	34.1^ab^	27.7^c^	37.6^a^	31.8^bc^	0.60
Arginine	32.6^ab^	32.9^a^	27.9^c^	33.2^a^	30.3^bc^	0.36
Total amino acids	433^ab^	442^a^	359^c^	453^a^	404^b^	4.8
Amino acids, g/kg CP	899	903	928	901	897	4.8
Available lysine	28.2^ab^	28.1^ab^	23.8^c^	29.8^a^	26.3^bc^	0.38
Available lysine, g/kg total lysine	840	825	863	794	830	15

Within a row, products not sharing a common superscript are significantly different (*P *< 0.05)

C-sSBM, solvent SBM made in China; C-eSBM, expanded SBM made in China; C-ff-eSB, full-fat expanded soybeans made in China; US-sSBM, United States solvent SBM; US-meSBM, United States mechanically extracted SBM; FA, fatty acids; CP, crude protein

^1 ^All values reported as g/kg of dry matter, except where indicated otherwise

^2 ^Standard error of the mean for n = 14. To obtain SEM for n = 5, multiply by 1.67; to obtain SEM for n = 2 multiply by 2.65

The C-ff-SB contained less (*P *< 0.01) CP and AA than the other products. C-sSBM and C-eSBM did not differ in content of CP or AA.

### Lysine chemical availability (FDNB-procedure)

C-eSBM and C-sSBM did not differ when chemically available lysine was expressed as a proportion of dry matter or as a proportion of total lysine (Table 2). Although lysine availability as a proportion of dry matter was greater for US-sSBM than for US-meSBM, they had similar lysine availability when expressed as proportion of total lysine; thus, the greater availability of lysine for US-sSBM than for US-meSBM was due to the higher total lysine content.

### *In situ *protein degradability of SBM

As expected, the proportion of N from the SBM remaining *in situ *decreased over time for all SBM products, with only a small amount of N remaining at 48 h except for C-ff-SB and US-meSBM (Table 3). No differences occurred in proportions of N remaining between C-sSBM and C-eSBM at 0, 4, 8, 24, or 48 h. The C-eSBM had significantly more N remaining at 16 h when compared with C-sSBM; this difference was not large enough to lead to differences in the predicted rates of *in situ *degradation or in the predicted RUP (Table 3).

**Table 3:** *In situ *protein degradability and intestinal availability of N from soybean meals (SBM) from China and the United States

	SBM made in China			United States SBM		
**Item**	**C-sSBM**	**C-eSBM**	**C-ff-eSB**	**US-sSBM**	**US-meSBM**	**SEM^1^**
n	5	14	2	2	2	
N remaining in situ, g/g						
0 h	0.909^a^	0.897^a^	0.768^b^	0.878^ab^	0.808^b^	0.016
4 h	0.691	0.710	0.630	0.669	0.691	
8 h	0.511^ab^	0.516^ab^	0.485^b^	0.439^b^	0.603^a^	
16 h	0.219^c^	0.293^bd^	0.300^bc^	0.218^cd^	0.444^a^	
24 h	0.112^c^	0.134^c^	0.223^b^	0.056^c^	0.356^a^	
48 h	0.014^b^	0.014^b^	0.072^ab^	0.006^ab^	0.113^a^	
In situ N kinetics						
B pool, g/g	0.934^a^	0.921^a^	0.785^c^	0.906^ab^	0.811^bc^	0.013
Kd, h^-1^	0.0857^a^	0.0762^a^	0.0697^a^	0.0942^a^	0.0386^b^	0.0031
RUP, g/g^2^						
Kp = 0.04/h	0.305	0.326	0.328	0.279	0.420	0.013
Kp = 0.06/h	0.392	0.414	0.402	0.361	0.499	0.015
Kp = 0.08/h	0.458	0.479	0.455	0.424	0.552	0.015
Three-step procedure^3^						
16-hour RUP, g/g	0.236^b^	0.263^b^	0.315^ab^	0.178^b^	0.434^a^	0.019
Intestinal digestion, g/g	0.832	0.827	0.846	0.832	0.843	0.0033
Total availability, g/g	0.199^b^	0.220^b^	0.269^ab^	0.148^b^	0.374^a^	0.016

Within a row, products not sharing a common superscript are significantly different (*P *< 0.05)

C-sSBM, solvent SBM made in China; C-eSBM, expanded SBM made in China; C-ff-eSB, full-fat expanded soybeans made in China; US-sSBM, United States solvent SBM; US-meSBM, United States mechanically extracted SBM; N, nitrogen; B pool, potentially degraded fraction; Kd, degradation rate of B pool; RUP, ruminally undegraded protein; Kp, ruminal passage rate

^1 ^Standard error of the mean for n = 14. To obtain SEM for n = 5, multiply by 1.67; to obtain SEM for n = 2 multiply by 2.65

^2 ^Calculated as: B × (Kp/(Kp+Kd))

^3 ^Calsamiglia and Stern [12]

### *In situ *kinetics of ruminal protein degradability of SBM

The insoluble N fraction (B pool) was similar among C-eSBM, C-sSBM, and US-sSBM (Table 3); however, the insoluble N was less for C-ff-eSB and US-meSBM than for the other products. The model-calculated values of insoluble N were generally comparable to the measured residual N at 0 h (Table 3), suggesting that the model appropriately predicted the soluble N pool. No differences were measured between C-eSBM and C-sSBM for Kd (Table 3). Also, no difference was found between C-sSBM, US-sSBM, and C-ff-eSB for Kd. The US-meSBM had the slowest (*P *< 0.01) rate of degradation among SBM sources.

No differences were found between C-eSBM and C-sSBM for their predicted RUP at the three passage rates (Table 3).

### Intestinal digestibility of RUP (three-step procedure)

The 16-h RUP concentrations differed (*P *< 0.05) among SBM products (Table 3). The US-meSBM had the highest 16-h RUP and US-sSBM had the least 16-h RUP. No difference was detected between the C-eSBM and C-sSBM for 16-h RUP or among SBM products for intestinal digestion of RUP determined by the 3-step procedure (Table 3).

Total availability of N calculated by multiplying the 16-h RUP by its intestinal digestibility differed (*P *< 0.01) among SBM products with the same pattern as for 16-h bypass (Table 3).

## Discussion

### Chemical composition of SBM

The lower proportion of long-chain fatty acids as C16:0 and a higher proportion as C18:1 for C-ff-SB and US-meSBM compared with other SBM products likely reflects that the composition of fatty acids in triglycerides differs from that of phospholipids and that more triglycerides remained in the higher fat products.

The lower CP and AA content of C-ff-SB reflects the dilution of these components by the greater amounts of fat. The CP and AA contents of C-eSBM were close to values for expanded SBM reported by Douglas and Parsons [7]. The CP and AA compositions of US-sSBM were comparable to National Research Council [15] values, and the CP and AA compositions of US-meSBM were comparable to those reported by Borucki Castro et al. [16].

### Lysine chemical availability

In the FDNB assay, unbound lysine residues react with FDNB to yield dinitrophenyl-lysine and are considered nutritionally available, whereas lysine residues in which the ε-amino group is bound will not react with FDNB and are not measured [17]. Faldet et al. [2] studied the effect of heat intensity and duration on lysine availability using the FDNB technique and a rat growth assay, and they reported that lysine availability was reduced with increasing heat intensity and duration. They also reported that the FDNB method provided accurate measures of available lysine when the result was compared with body weight gain of rats.

The expansion of SBM prior to fat extraction had no effect on lysine availability in the set of Chinese samples that we evaluated, indicating that heat during the expansion process was not high enough to cause protein damage. The coefficient of variation (standard deviation/mean) for available lysine among C-eSBM samples was small (0.05), indicating that there was not large variation among sources.

### *In situ *kinetics of ruminal protein degradability of SBM

The process of denaturation and cross-link formation during heating reduces protein solubility and makes protein less available for rumen degradation by rumen micro-organisms [18]. The reduction of ruminal protein degradability by heating has been reported by many researchers [19-22]. Although we had expected that the additional heat from the expansion process might reduce ruminal protein degradability, our results indicate that the C-eSBM samples did not undergo enough additional heating during the expansion process to affect nutritive value of the protein for ruminants. In general, the temperature used in the expanding process is around 120°C, and the pressure can be automatically controlled. In contrast, during extrusion processes that impact degradability of soybean proteins, the temperature often reaches 165°C. Prestløkken [23] demonstrated that expansion of SBM with an outlet temperature of 129°C increased the RUP content. The lack of difference between C-eSBM and C-sSBM for ruminal protein degradability might indicate either that temperatures less than 129°C were used during the expansion process for C-eSBM or that expansion of soybeans before oil extraction impacts the soybean proteins less than expansion of SBM. Regardless, expansion did not have significant effects on RUP for the samples we evaluated.

The US-meSBM had numerically greater RUP than other products due to slower rates of ruminal degradation of protein; they also had a somewhat larger soluble N pool (1-B pool) than the other SBM, which would decrease the predicted RUP. Awawdeh et al. [11] found numerically greater soluble N in mechanically extracted SBM compared with solvent-extracted SBM. The greater soluble N of US-meSBM could be explained by destruction of some of the polypeptide bonds in SBM proteins as a result of heat; Wolf [24] reported that temperatures greater than 100°C could increase protein solubility as a result of destruction of polypeptide bonds. However, the higher RUP for US-meSBM indicates that much of the protein underwent denaturation that reduced ruminal protein degradability by rumen micro-organisms. The estimated RUP of US-sSBM at passage rate of 0.08/h was similar to the estimated RUP of 0.43 reported by the National Research Council [15].

### Intestinal digestibility of RUP (three-step procedure)

Heat treatment can improve intestinal digestibility because of the destruction of the anti-trypsin factors by heat [25]. Aldrich et al. [26] found that the anti-trypsin activity was reduced linearly with increasing temperature during extrusion of soybeans. Solanas et al. [27] reported that extrusion of SBM increased the intestinal digestibility of the bypass protein; however, excessive heat reduces protein digestibility and causes destruction to essential AA, especially lysine, which is considered the most sensitive AA to heat [28]. Demjanec et al. [20] investigated the effect of heating SBM at 165°C for 0, 75, 150, 180, or 210 min on N flow to and digestibility in the small intestine of wethers fitted with duodenal and ileal canulas. Intestinal digestion of SBM-N increased with heating times up to 150 min, then declined as heating time was extended; this demonstrates both the beneficial effects of moderate heating and the negative effects of overheating.

The lack of difference among treatments for intestinal digestibility along with the small standard error for intestinal digestion suggests that products were quite similar and that none experienced significant heat damage. Given the lack of treatment difference for intestinal digestion, it is not surprising that the pattern for total N availability was predominantly dictated by 16-h RUP rather than by intestinal digestion.

### Summary

Our data demonstrated no large differences between C-eSBM and C-sSBM in nutrient composition, ruminal protein degradability, or intestinal protein digestibility indicating that heating during the expansion process was not enough to affect the nutritive value of SBM for ruminants. Although the expansion process may improve the oil extraction process, our *in vitro *data suggest that effects of expansion on the resulting SBM will not require consideration when formulating ruminant diets. If more extensive heating was applied with the goal of altering SBM proteins, then further research may be warranted to evaluate new processes.

## Competing interests

The authors declare that they have no competing interests.

## Authors' contributions

EAE participated in design of the research, carried out the experiment, analyzed data, and drafted the manuscript. ECT designed the research, coordinated the research, conducted statistical analyses, and had primary control of the final content of the manuscript. ZJC participated in design of the research, collected soybean samples from China, and contributed to preparation of the manuscript. AMN and MEAN participated in design of the research and contributed to preparation of the manuscript. All authors read and approved the final manuscript.
